# Rare case of umbilical urachal sinus mimicking infected umbilical abscess: A case report

**DOI:** 10.1002/ccr3.5598

**Published:** 2022-03-16

**Authors:** Hossein Torabi, Kasra Shirini, Rona Ghaffari

**Affiliations:** ^1^ Department of General Surgery Poursina Medical and Educational Center Guilan University of Medical Sciences Rasht Iran; ^2^ Department of General Surgery Iran University of Medical Science Tehran Iran; ^3^ Department of General Surgery, Poursina Medical and Educational Center Guilan University of Medical Sciences Rasht Iran

**Keywords:** abdominal pain, umbilical abscess, umbilical discharge, umbilical pilonidal sinus, umbilical urachal sinus

## Abstract

Incomplete obliteration of urachus during the fetal period can lead to urachal abnormalities. One of these abnormalities is the urachal sinus that can be asymptomatic, or it can be symptomatic by becoming infected or being malignant, and it can mimic other diseases' symptoms. Although it is rare in adults, it should be considered a significant differential diagnosis in patients with abdominal pain or umbilical discharge. This article presents a young patient with urachal sinus mimicking umbilical pilonidal sinus symptoms.

## Graphical abstract

Although umbilical urachal sinus is a rare condition in adults, it should be considered as a significant differential diagnosis in patients with abdominal pain or umbilical discharge due to the development of infection or malignancy.

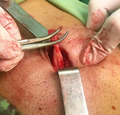

## 1 INTRODUCTION

If urachus obliteration occurs incompletely during the fetal period, it can lead to urachal abnormalities.^1^ Urachal abnormalities are rare in adults and approximately 1 per 50.000 hospitalizations.^2^, ^3^ There are five different types of urachal abnormalities. One of them is the urachal sinus, which drains proximally into the umbilicus, accounts for 15% of cases.^2^, ^4^ The symptoms of this disease are nonspecific and can have different manifestations, such as omphalitis.^1^, ^5^ The main diagnostic way is using ultrasonography which can diagnose 77% of patients.^6^ There are different ways to treat infected urachal sinus, but the primary treatment is surgery to eradicate the remaining urachal tissue throughout its length due to the risk of developing infections or carcinoma.^7^, ^8^ Due to the rarity of this condition, nonspecific symptoms, and side effects, the diagnosis is a significant challenge. In this article, a young male with urachal sinus presented with a clinical presentation mimicking an umbilical pilonidal cyst.

## 2 CASE PRESENTATION

**FIGURE 1:**
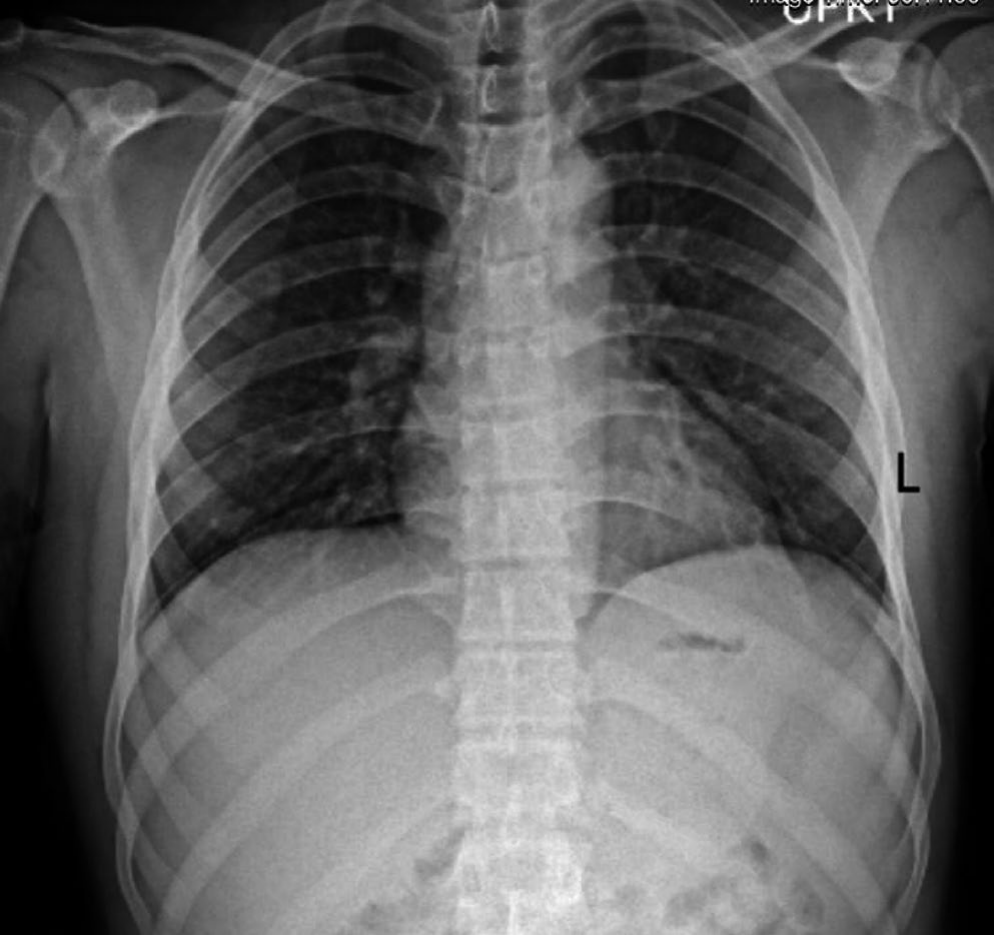
Upright Chest X‐ray

**FIGURE 2:**
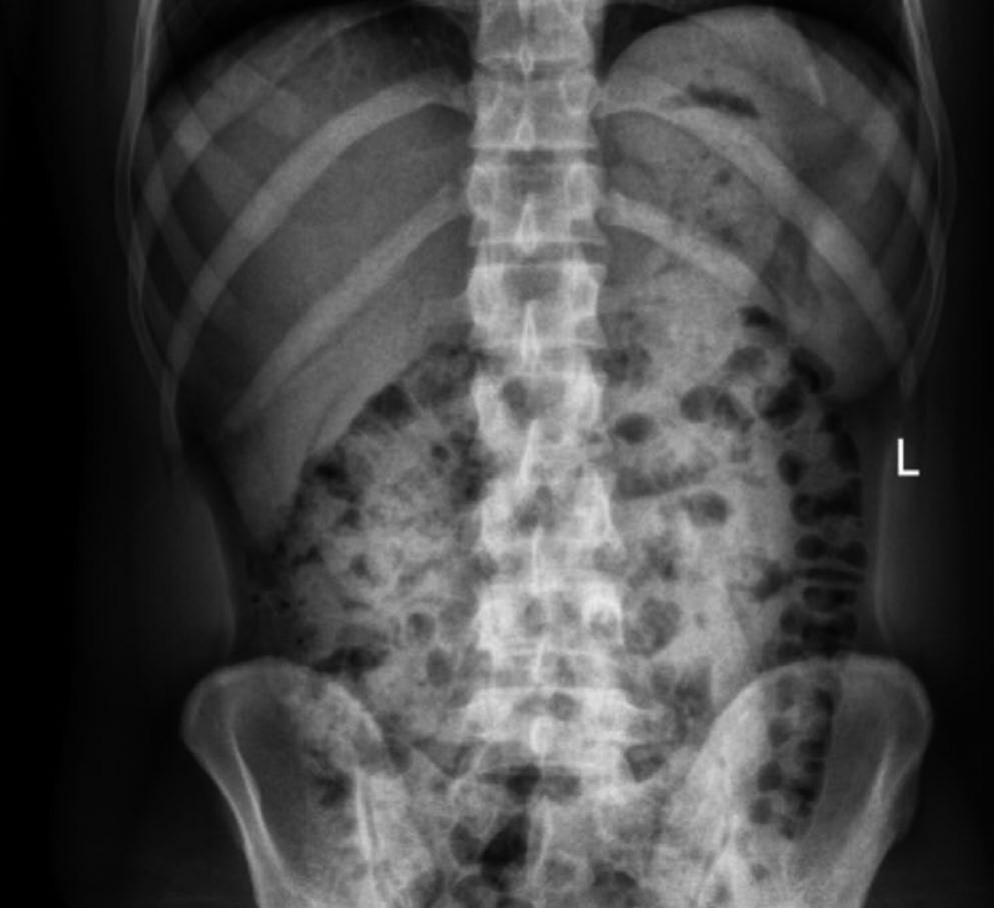
Upright abdominal X‐ray

**FIGURE 3:**
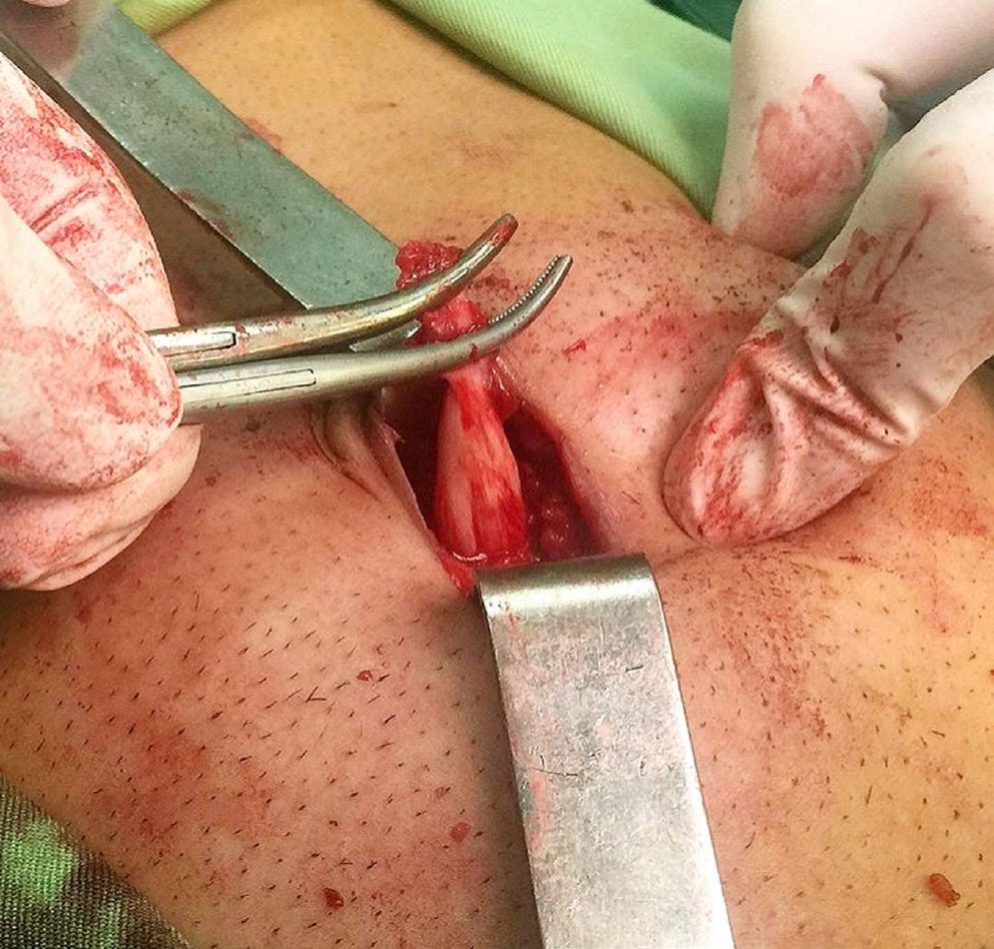
Umbilical urachal sinus and obliterated urachal ligament, which was fund during surgery

A 28‐year‐old man presented to the surgical clinic of Poursina Hospital Medical Center, in Rasht, Iran, in September 2021, with complaints of pain and discharge of stinky pus and blood from the umbilical had suddenly started about three weeks before the patient's attendance at the hospital. He claimed that he attended a doctor at first and was prescribed clindamycin and ciprofloxacin tablet for ten days with suspicion of infected umbilical pilonidal sinus, but the medications did not have any positive effect, the discharge continued. The past medical history was unrevealing. The patient had a deep umbilicus, dense hairy abdomen, a tuft of hair was seen in his umbilical area, the umbilical area was erythematosus in appearance, and it was tender, but other physical examinations did not include abnormal findings. His vital signs were in the normal range. He was asked to do an upright chest X‐ray and an upright abdominal X‐ray, as shown in Figures 1 and 2, and the images were unrevealing. He was asked to do abdominal ultrasonography. The ultrasonography report showed a hypoechoic area measuring 23 × 6 mm, which could be a collection. The blood test analysis presented an average level of erythrocyte sedimentation rate (ESR) = 9 (usually should be under 15 in males) and leukocytosis (white blood cells [WBC] = 15,800 g/dl with a neutrophilia ratio of 74%). Other factors were within the standard ratio. So, due to the ineffectiveness of the drugs and the continuation of the patient's symptoms, the patient underwent surgery with the suspicion of umbilical pilonidal sinus. A circumferential incision was performed around the umbilical. Inconceivably, urachal umbilical sinus was seen, which was connected by obliterated urachus to the apex region of the bladder wall, as can be seen in Figure 3. After total resection of connection between the obliterated urachus and gallbladder wall and repairing the gallbladder wall, the umbilicus was placed in its initial place, and the incisions were closed. The patient had a proper recovery. On postoperative Day one, the patient was discharged from the surgical service in good general condition. After three months of follow‐up, he did not have any symptoms of infection or discharge.

## 3 DISCUSSION

The urachus is a vestigial of embryonic structures, which remains a fibrous cord between the anterior transversalis fascia and the peritoneum posterior. This fibrous tissue attaches the umbilicus to the bladder.^1^ The incidence of urachal abnormalities is higher in children than adults. It is rare in adults with a ratio of 2%, and it is twice as likely to occur in males as in females.^2^ There are five different types of urachal abnormalities included that patent urachus (50%), urachal cyst (30%), urachal sinus (15%), vesicourachal diverticulum (3%–5%), and alternating sinus. Urachal sinus can be asymptomatic until infected or malignant.^7^, ^8^ Infected urachal sinus can present with nonspecific symptoms such as lower abdominal pain, fever, redness around the umbilicus, and umbilical discharge, or even it can cause sepsis. During the physical examination, infraumbilical mass under the skin may be felt.^9^ In some cases, the urachal sinus can be infected by Escherichia coli, Enterococcus faecium, Proteus, Streptococcus viridans, and Fusobacterium, leading to urachal sinus abscess causing abdominal pain and umbilical discharge.^2^, ^4^ Urachal sinus could be malignant, including less than 5% of bladder cancers, and the most common type is adenocarcinoma.^10^ The most common imaging method used to investigate this disease is ultrasonography, which some reported showed 77%–90% diagnostic accuracy of ultrasonography for urachal anomalies, especially in children and young adults. Although, other imaging methods such as computed tomography (CT), sinography, or magnetic resonance imaging (MRI) can be used if ultrasonography does not have the necessary accuracy to diagnose the anomaly.^6^, ^11^, ^12^

In this case report, a young male with a deep umbilicus, dense hairy abdomen, a tuft of hair which was seen in his umbilical area, and erythematosus umbilical area presents with the complaint of discharge of pus and blood presented to the surgical clinic. All the symptoms could be considered as an umbilical pilonidal sinus.

Umbilical pilonidal sinus is one of the types of pilonidal sinus disease, which is defined as a miscellaneous clinical presentation from cysts and sinuses containing hair without any symptoms to large abscess with different symptoms that can happen in various parts of the body.^13^

There are two main treatment methods for umbilical pilonidal sinus, including conservative and surgical methods, and choosing the proper method depends on medical team preferences and the patient's condition.^13^, ^14^ Due to the lack of proper effect of antibiotics and the patient's condition, and due to the preference of the surgical team according to the confirmation of the diagnosis by the ultrasonography report and without any further imaging, the patient underwent surgical treatment.

So, due to the rare possibility of the urachal sinus in adults, side effects of this disease such as infection or malignancy, and nonspecific clinical findings, it should be considered a differential diagnosis in patients presenting with lower abdominal pain or umbilical discharge.

## 4 CONCLUSION

Urachal sinus is a type of urachal abnormalities caused by incomplete urachal obliteration. This disease is rare in adults, and it is more in males. However, as our study showed this disease can be asymptomatic until it becomes infected, malignant, and subsequently mimic other diseases' symptoms such as appendicitis, urinary tract disease, or umbilical pilonidal sinus. So, because of the misdiagnosis and probability of urachal sinus malignancy or infection, it is crucial to accurately identify and diagnose this disease by further imaging investigations and consider it an important differential diagnosis in patients with abdominal pain or umbilical discharge, even in adults.

## CONFLICT OF INTEREST

The authors certify that there is no conflict of interest with any financial organization regarding the material discussed in the manuscript. The patient has consented to the submission of the case report for submission to the journal.

## AUTHOR CONTRIBUTIONS

All authors contributed equally to the manuscript and read and approved the final version of the manuscript.

## CONSENT

Written informed consent was obtained from the patient to publish this report in accordance with the journal's patient consent policy.

## Data Availability

The data that support the findings of this study are available on request from the corresponding author. The data are not publicly available due to privacy or ethical restrictions.
